# Supplementary material for: Perceived Barriers to a Healthy Diet and Factors Associated With Unhealthy Food Consumption in Steatotic Liver Disease
Source: Gastro Hep Adv. 2026 May 27;5(9):101024. doi: 10.1016/j.gastha.2026.101024 (PMC13348045; doi:10.1016/j.gastha.2026.101024)
Supplement: Supplementary Materials [file mmc1.pdf]

Perceived Barriers to a Healthy Diet and Factors Associated with Unhealthy Food Consumption in Steatotic Liver Disease

Serena N Gilmore, Catherine Magee, and Mandana Khalili

Table of contents

Table of Contents

SUPPLEMENTAL TABLE 1. 2

SUPPLEMENT TABLE 2.......... 4

Table S1. Frequency of consumption of unhealthy food categories survey questionnaire

Question	Response choices
**How often do you eat rice/bread/tortillas?**	None<br>1 time per week<br>2-3 times per week<br>4-5 times per week<br>6 or more times per week
**On average, how often do you eat fast food?**	Not at all<br>One time per month<br>2-3 times per month<br>4-5 times per month<br>6 or more times per month
**How often do you eat fried food? (chow mein, fried chicken, chips, french fries, chicharrones, tostadas, refried beans)**	None<br>1 time per week<br>2-3 times per week<br>4-5 times per week<br>6 or more times per week
**How often do you have sweets such as baked goods, candy, and ice cream?**	None<br>1 time per week<br>2-3 times per week<br>4-5 times per week<br>6 or more times per week
**How often do you drink sugar-sweetened beverages per week? (ie: soda, boba, agua fresca,<br>juice, sports drink, etc)**	None<br>1 time per week<br>2-3 times per week<br>4-5 times per week<br>6 or more times per week
**How many cups of tea (black or green) did you typically drink in the past year? (one cup oftea equals 8 ounces, hot or cold)**	None<br>Occasionally (less than 1 per day)<br>1 per day<br>2 per day<br>3 or 4 per day<br>More than 4 per day
**How many cups of coffee did you typically drink in the past year? (one cup of coffee equals 8 ounces. Count one espresso as one cup regardless of size)**	None<br>Occasionally (less than 1 per day)<br>1 per day<br>2 per day<br>3 or 4 per day<br>More than 4 per day

**Do you drink your coffee or tea with sugar?**	Yes<br>Type of sweetener:<br>Sugar (raw/brown/refined etc.)<br>Artificial sweetener (Splenda, Sweet'N Low, Equal, Stevia etc.)<br>Sweetened Creamer (Coffee Mate, Delight, etc)<br>Honey/agave<br>Number of packets or spoons: _____________<br>No

Table S2. Survey question regarding barriers to eating healthy foods

**Level of Influence**	**What barriers (if any) do you experience in following your provider's recommendations for healthy eating?**
Societal	-Difficulty buying healthy foods<br>-Cost of healthy foods<br>-Difficulty buying healthy foods
Community	-In my culture we eat certain foods, and it is important for me to eat this way with other members of my community
Interpersonal	-Family members do not eat healthy, so difficult for me to change my diet<br>-In my family, we eat certain foods, and it is important for me to eat this way with my family
Individual	-Feeling nervous or anxious about cooking<br>-Not motivated to eat healthy foods/exercise<br>-Do not know how to cook healthy foods<br>-Do not believe healthy diet/exercise are important<br>-Never been told by doctor to eat healthier/exercise more<br>-Some else cooks for me<br>-Do not like the taste of healthy foods<br>-Lack of time to eat healthy foods/exercise
