# Supplementary material for: Perceived Barriers to a Healthy Diet and Factors Associated With Unhealthy Food Consumption in Steatotic Liver Disease
Source: Gastro Hep Adv. 2026 May 27;5(9):101024. doi: 10.1016/j.gastha.2026.101024 (PMC13348045; doi:10.1016/j.gastha.2026.101024)
Supplement: Extended PDF [file mmc2.pdf]

Gastro Hep Advances 2026;5:101024

ORIGINAL RESEARCH—CLINICAL

Perceived Barriers to a Healthy Diet and Factors Associated With Unhealthy Food Consumption in Steatotic Liver Disease

Serena N. Gilmore,[1,2,3] Catherine Magee,[2] and Mandana Khalili[1,2,3]

[1]*School of Medicine, University of California, San Francisco, San Francisco, California;* [2]*Division of Gastroenterology and Hepatology, Zuckerberg San Francisco General Hospital and Trauma Center, San Francisco, California; and* [3]*Department of Medicine, University of California, San Francisco, San Francisco, California*

BACKGROUND AND AIMS: Dietary modification is important to steatotic liver disease (SLD) management. We aimed to determine barriers to a healthy diet and factors associated with unhealthy eating in a diverse and socioeconomically disadvantaged population. **METHODS:** For this cross-sectional study, 344 adults with SLD at hepatology clinics of a safety-net health-care system were surveyed between February 2020 and February 2024. Clinical data were collected using electronic medical records. An unhealthy diet score was calculated based on the frequency of unhealthy food consumption, and its median defined the cutoff for unhealthy diet outcome. Logistic regression evaluated the relationship between barriers to healthy eating and unhealthy food categories. Multivariable model adjusting for age, sex, race, obesity, and exercise evaluated factors associated with unhealthy diet score. **RESULTS:** Participant demographics were median age 53 years, 58% female, and 57% Latino. Majority (60%) were obese and 25% had heavy alcohol use. Common barriers were lack of motivation, cost, and lack of healthy cooking knowledge. Carbohydrates and sugar-sweetened beverages consumption were associated with barriers at multiple levels of influence. Lack of motivation to eat healthy foods and/or exercise was significantly associated with increased consumption of fast foods (odds ratio [OR] 1.79) and fried foods (OR 2.72). On adjusted multivariable analysis, age (per decade, OR 0.69) and any exercise (OR 0.52) was associated with lower unhealthy diet scores (all $P < .05$). **CONCLUSION:** Promoting a healthier diet is especially important in younger age groups and those with lower physical activity in SLD. Dietary educational interventions and culturally tailored healthy cooking practices is critical to enhancing confidence and motivation for dietary change.

Keywords: Vulnerable Population; Health Disparity; Metabolic Dysfunction–Associated Steatotic Liver Disease; Alcohol-Related Liver Disease; MetALD

Introduction

Steatotic liver disease (SLD) is characterized by different subtypes based on etiology and includes metabolic dysfunction–associated steatotic liver disease, alcohol-related liver disease, or both, termed metabolic and alcohol-Associated liver disease.[1,2] The prevalence of SLD, with metabolic dysfunction–associated steatotic liver disease being the leading subtype, has increased over the years, with an estimated prevalence of 34% in the United States and over 37% worldwide.[2–4] Furthermore, the prevalence of SLD differs based on race/ethnicity, sex, and socioeconomic status (SES), with men, Hispanic Americans, and vulnerable and socioeconomically disadvantaged populations being disproportionately affected.[5]

The primary management of SLD consists of lifestyle changes, notably a healthier diet and exercise. The Mediterranean diet, high in fruits, vegetables, fiber, omega-3, and omega-6 fatty acids, has been found to be beneficial for those with SLD.[6–8] It is recommended to have a higher consumption of these foods and a lower consumption of simple carbohydrates, sugars, and saturated fats.[9,10] Food insecurity is a term describing decreased access to healthy foods, and this has been associated with poorer liver health[5]; however, additional barriers to the consumption of a healthier diet for liver health have not been more widely studied. These barriers could be at the community level, such as cultural values; the interpersonal level, such as family dynamics; and the individual level, such as personal knowledge about food nutrition.[11,12] Determining these barriers is especially important for populations more affected by SLD, specifically racial and ethnic minorities and people of low SES, majority of whom received care within safety-net patient populations. These patients are disproportionately affected by SLD health disparities,[13] have complex social and health needs, and in addition to low SES, experience language, and cultural barriers to health care.

In this study, using the National Institute on Minority Health and Health Disparities framework,[14] our primary aims and secondary exploratory aims were as follows: (1) to describe perceived barriers among a diverse, low SES SLD population receiving care at a safety-net health-care

Abbreviations used in this paper: API, Asian/Pacific Islander; CI, confidence interval; SES, socioeconomic status; SLD, steatotic liver disease.

Most current article

© 2026 The Author(s). Published by Elsevier Inc. on behalf of American Gastroenterological Association Institute. This is an open access article under the CC BY license (http://creativecommons.org/licenses/by/4.0/).
2772-5723
https://doi.org/10.1016/j.gastha.2026.101024

system at the personal, interpersonal, community, and societal levels of influence[15] and (2) to evaluate factors associated with consumption of an unhealthy diet.

Patients and Methods

Study Population

This study enrolled 344 adult patients (≥ 18 years of age) with SLD receiving care at hepatology clinics at an urban safety-net hospital, Zuckerberg San Francisco General Hospital, serving socioeconomically and medically disadvantaged populations of San Francisco between February 19, 2020, and February 28, 2024. Inclusion criteria included a diagnosis of SLD, as defined by the presence of steatosis on liver biopsy or imaging (eg, liver ultrasound, magnetic resonance imaging/computed tomography abdomen and pelvis) and documentation by a liver specialist of either metabolic-dysfunction and/or alcohol-associated etiologies.[16] A medical or psychiatric condition that prevented the completion of study activities excluded participants from the study. This study was approved by the Institutional Review Board of the University of California, San Francisco, and Zuckerberg San Francisco General Hospital.

Study Design and Data Collection

For this cross-sectional study, following informed consent, patients completed the following questionnaires: (1) sociodemographic data; (2) modified food frequency questionnaire; (3) exercise intensity and duration; and (4) perceived barriers to a healthy diet. The perceived barriers to a healthy diet questions were derived from questionnaire instrument that was developed using the Health Behavior Framework with input from expert hepatologists and behavioral scientists experienced in health behavior change research and information from published studies in patients with liver disease.[16–18] Clinical data were collected using self-report and electronic health record review. Participants were compensated $25 for participating in the informational session and answering all survey material. For non-English speakers, all surveys were translated into Spanish, the most prevalent language spoken outside of English in our population, and certified medical interpreters were used for other languages as needed.

Data Definitions and Measures

Sociodemographic information included age, sex, race/ethnicity, and social determinants of health.[19] Social determinants of health were highest educational level attainment, employment status, annual income, housing stability, number of people in the household, primary language, and English language fluency. The National Institute on Alcohol Abuse and Alcoholism questionnaire was used to categorize alcohol use.[19]

A survey was designed that included questions on patients' frequency of consumption of unhealthy foods using modification of the Food Frequency Questionnaire that focused on frequency of consumption of fried foods, fast foods, sweets or desserts, carbohydrates (rice, bread, and/or tortillas), and sugar-sweetened beverages (Supplementary Table 1). There were 5 response options for frequency of consumption of carbohydrates, fried foods, sweets, and sugar-sweetened beverages as follows: none, once weekly, 2–3 times weekly, 4–5 times weekly, or 6 or more times weekly. For fast food, the 5 response options were none, once monthly, 2–3 times monthly, 4–5 times monthly, or 6 times monthly. Each response was assigned a numerical value, with "0" representing none and "4" representing maximum frequency of consumption for these 5 unhealthy food categories. Due to the data not being normally distributed, we dichotomized the score for each of the 5 unhealthy food groups using the observed median values as follows: consumption of carbohydrates <3 or ≥ 3, fast foods <1 or ≥ 1, fried foods <1 or ≥ 1, sweets <1 or ≥ 1, and sugar-sweetened beverages <1 or ≥ 1. A total unhealthy diet score, which will be referred to as unhealthy diet score, for each participant was also calculated by adding the numerical score from each of the 5 unhealthy food groups consumed for a possible maximum unhealthy diet score of 20. Given that our questionnaire focused on frequency of consumption rather than quantity, quantitative dietary guidelines could not be utilized for categorizing unhealthy diet. Therefore, similar to prior studies,[20] this unhealthy diet score was dichotomized using the observed median value, with <7 representing a healthier diet and ≥ 7 representing an unhealthy diet.

To support the choice of dichotomization threshold for the unhealthy diet score using the median value of 7 as the dichotomization threshold of "healthier" and "unhealthy" groups, a latent profile analysis (LPA) of the 5 ordinal unhealthy diet survey items (with 0–4 ratings) was performed to determine the best-fitting two-cluster model based on Bayesian information criterion. LPA is a type of finite mixture modeling that identifies hidden subpopulations (latent classes) within a population based on numerical indicators. The best-fitting two-cluster model based on Bayesian information criterion consisted of 83% of the cohort in group 1% and 17% in group 2. Group 1 ("unhealthy") had median (Q1-Q3) 8 (6–11) unhealthy diet score, while group 2 ("healthier") had median (Q1-Q3) 6 (5–7). The unhealthy diet score that fell between the medians of the 2 derived clusters was 7, which corresponds to the median score of the full cohort and to the upper quartile value of the "healthier" group, supporting the choice of dichotomization threshold at 7.

We assessed physical activity duration and intensity using a questionnaire that was developed based on clinical recommendations and categorized the presence and absence of any reported exercise.[19] Patients' barriers to lifestyle modifications were assessed and categorized using a modified National Institute on Minority Health and Health Disparities framework into levels of individual, interpersonal, community, and societal (Supplementary Table 2).[15,21]

Electronic health record review was used to collect clinical history and laboratory data. Body mass index was categorized and race-adjusted as follows: normal <25 kg/m^2 (<23 kg/m^2 if Asian/Pacific Islander [API]), overweight 25 to 29.9 kg/m^2 (23–27.4 kg/m^2 if API), and obese ≥30 kg/m^2 (≥27.5 kg/m^2 if API).[22,23] Advanced fibrosis/cirrhosis was defined as presence of contour nodularity of the liver with or without splenomegaly or venous collaterals on imaging, magnetic resonance elastography liver stiffness measurement >4.5 kPa, or a histologic fibrosis stage of F3-4. Participants' comorbidities, including diabetes, hypertension, hyperlipidemia, anxiety, and depression, were also captured.

Statistical Analysis

Participant data and characteristics were summarized using percentage and median (interquartile range). The relationship between each perceived barrier reported and consumption of each of the 5 unhealthy food categories was assessed using logistic regression. Prespecified covariates were selected a priori based on literature review and clinical knowledge and considered in univariable and multivariable logistic regression models for the outcome of unhealthy diet score. Age (per decade), sex, race/ethnicity (Latino vs non-Latino), body mass index category (obesity vs normal weight and overweight vs normal weight), and any exercise (vs none) were included in the multivariable model, and variable selection was used for all other potential risk factors. To prevent overfitting the models, the number of covariates allowed in the multivariable model was restricted to a maximum of 1 covariate level per 5 outcome events, where a covariate level is defined as 1 continuous variable or 1 category of a categorical variable.[24] Based on the number of variables, stepwise forward variable selection with an entry criterion of $P < .05$ in the unadjusted models for candidate risk factors, along with clinical judgment was used for variable selection so that the model was not overfit. Odds ratios (ORs) and 95% confidence intervals (CIs) were reported from all models. Hypothesis tests were 2-sided, and the significance threshold was set at $P < .05$. Statistical analysis was performed using Stata (version 16; StataCorp LLC, College Station, TX) and the R (version 4.3.2; R Foundation for Statistical Computing, Vienna, Austria) package mclust was used for the LPA.[25]

Table 1. Participant Characteristics

Characteristic (N = 344)[a]	Value
Age (median, interquartile range)	53 (42,62.5)
Female sex, n (%)	198 (58)
Race/ethnicity, n (%)	
Asian	90 (26)
Black	11 (3.2)
Hispanic	197 (57)
White	35 (10)
Other	11 (3.2)
Primary language, n (%)	
English	78 (23)
Spanish	178 (52)
Cantonese	51 (15)
Vietnamese	3 (0.87)
Other	34 (9.9)
Foreign born, n (%) (n = 342)	280 (81)
Fluent in English, n (%) (n = 331)	110 (32)
Education level at high school or less, n (%) (n = 334)	214 (64)
Unemployed in past year, n (%) (n = 339)	218 (64)
Annual income of <30,000, n (%) (n = 234)	181 (77)
Stable housing, n (%) (n = 329)	305 (93)
Household members of 3 or more, n (%) (n = 338)	191 (57)
Race-based BMI[b], n (%) (n = 324)	
Normal	31 (9.6)
Overweight	99 (31)
Obese	194 (60)
Comorbidities [N (%) (N = 324)]	
Diabetes	126 (37)
Hypertension	143 (42)
Hyperlipidemia	154 (45)
Anxiety	32 (9.3)
Depression	77 (22)
Alcohol intake, n (%) (n = 338)	
None	211 (62)
Moderate	43 (13)
Heavy	84 (25)
Any exercise, n (%) (n = 244)	164 (67)
Advanced fibrosis/cirrhosis, n (%)	76 (22)

BMI, body mass index.
[a]Unless otherwise specified in the table.
[b]Race-based BMI, categories: normal weight <25 kg/m2 (<23 kg/m^2 for Asian), overweight 25–29 kg/m^2 (23–27.4 kg/m^2 for Asian), and obese >30 kg/m^2 (≥27.5 kg/m^2 for Asian).

Results

Characteristics

The characteristics of the 344 participants are reported in Table 1. Participants had a mean age of 53 years (interquartile range 42, 62.5). There was a higher proportion of females (58%) and those of Hispanic ethnicity (57%). Less than a third of the participants were fluent in English (32%), less than 40% had an education above high school level, and a majority were unemployed and had an annual income of less than $30,000 (64% and 77%, respectively). As expected, obesity, hypertension, hyperlipidemia, and diabetes were prevalent.

The Frequency of Consumption of Unhealthy Food Categories

Figure 1 summarizes the participants' responses to the consumption of the 5 unhealthy food categories, along with consumption of tea and coffee. The majority (58%) of patients consumed fast foods less than 2 times per month. Fried foods, sweets, and sugar-sweetened beverages were consumed at 2 times or more per week at 33%, 34%, and 36%, respectively. With respect to carbohydrates, the frequency of weekly consumption was greater than any reported unhealthy food category (28% at 2 to 3 time per week and 55% at 4 or more times per week). More than 65% of participants consumed tea and 78% consumed coffee (5.5% consumed 3 or more cups of coffee per day), with 56% of the study participants indicating that they put sugar or sweetener in their coffee or tea (among those, 54% sugar, 27% artificial sweetener, 15% sweetened creamer, and 12% honey/agave).

Factors Associated With Unhealthy Food Score

On univariable analysis, older age (per decade, OR 0.71, 95% CI 0.59–0.84, $P < .001$), female sex (OR 0.61, 95% CI 0.40–0.90, $P = .03$), diagnosis of diabetes (OR 0.56, 95%

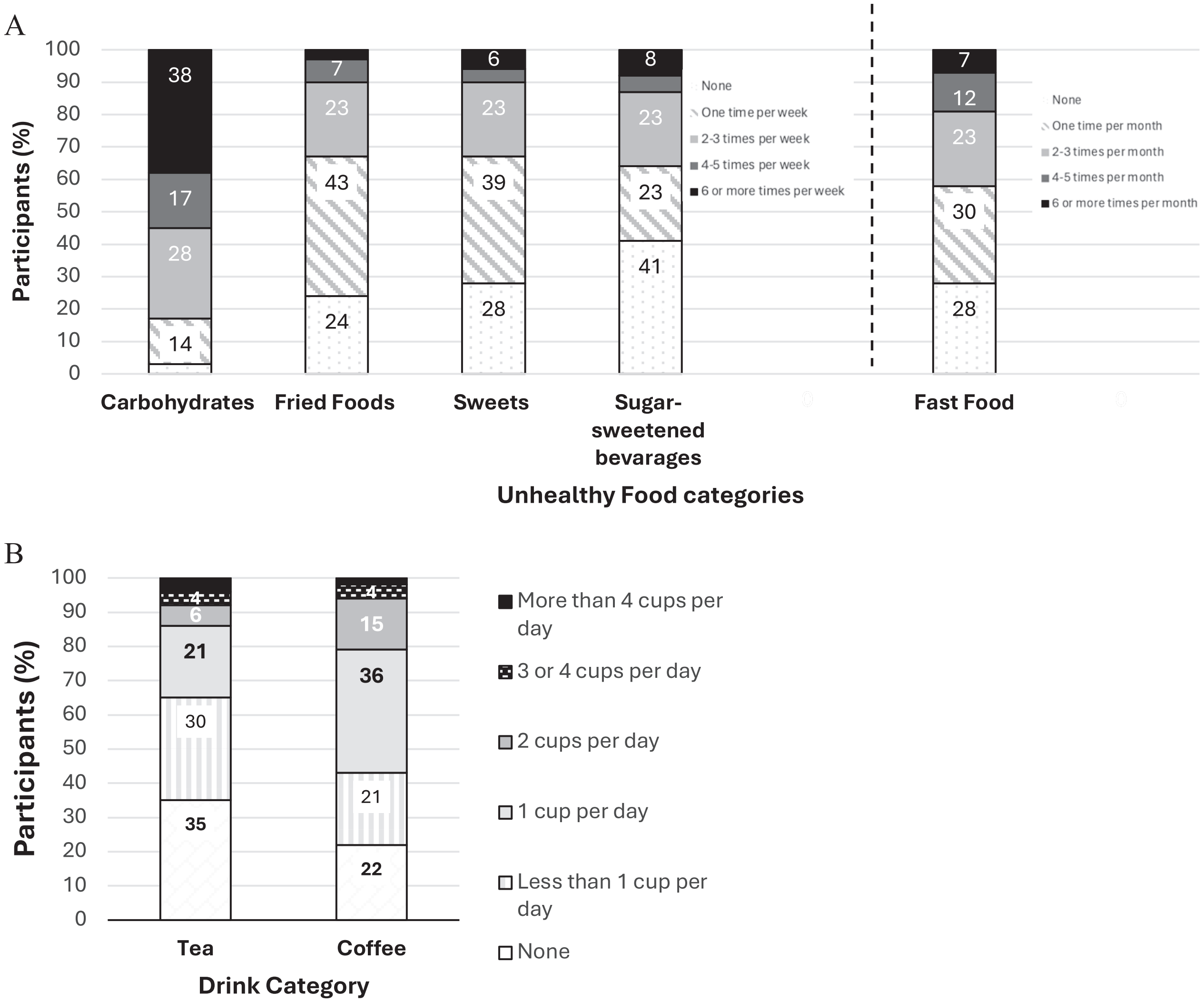

Figure 1. Frequency of consumption of food categories. (A) The frequency of consumption of carbohydrates, fast foods, sweets, sugar-sweetened beverages, and fast food. (B) The frequency of intake of tea and coffee.

CI 0.36–0.80, $P = .01$), and any exercise (OR 0.56, 95% CI 0.33–0.93, $P = .03$) were associated with a healthier diet (Table 2). English language fluency (OR 1.71, 95% CI 1.07–2.70, $P = .03$) was associated with an unhealthy diet. On multivariable analysis adjusted for age, sex, race/ethnicity, obesity, and any exercise, age (per decade, OR 0.69, 95% CI 0.56–0.84, $P < .001$) and any exercise (OR 0.52, 95% CI 0.30–0.91, $P = .02$) were associated with a healthier diet (Table 2).

Perceived Barriers and Consumption of Unhealthy Food Categories

The percentage of participants with a reported perceived barrier is seen in Figure 2. The relationships between reported perceived barriers across levels of influence (individual, interpersonal, community, and societal) and consumption of unhealthy food categories are presented in Table 3. The most commonly reported perceived barriers were not being motivated, cost of healthy foods, and not knowing how to cook healthy foods. There were perceived barriers identified at 3 levels of influence that were associated with carbohydrates (ie, community, interpersonal, and individual levels) and sugar-sweetened beverages (ie, societal, interpersonal, and individual levels). Whereas both fried food and fast food consumption was associated with multiple individual level barriers, fried food consumption was also influenced by community level barriers. Notably, lack of motivation to eat healthy foods and/or exercise was associated with both increased consumption of fast foods and fried foods (OR 1.79, 95% CI 1.01–3.17, $P < .05$ and OR 2.72, 95% CI 1.31–5.63, $P = .007$, respectively). Consumption of sweets was not significantly associated with any of the reported barriers. However, lack of healthy food consumption by family was associated with 2.29 higher odds of consuming sweets ($P = .06$), though this did not reach statistical significance.

Table 2. Univariable and Multivariable Logistic Regression for Association Between Participant Characteristics and Unhealthy Diet Score

Characteristic	Univariable model			Multivariable model		
	Odds ratio	95% CI	*P* value[a]	Odds ratio	95% CI	*P* value[a]
Age (per decade)	**0.71**	**0.59–0.84**	**< .001**	**0.68**	**0.56–0.84**	**< .001**
Sex	**0.61**	**0.40–0.90**	**.03**	0.62	0.38–1.00	.05
Latino (vs non-Latino)	0.77	0.50–1.10	.25	0.60	0.36–1.00	.05
BMI category (vs normal weight)						
Overweight	1.03	0.46–2.0	.93	0.90	0.38–2.10	.80
Obesity	1.52	0.71–3.2	.28	1.19	0.53–2.67	.68
Any exercise (vs none)	**0.56**	**0.33–0.93**	**.03**	**0.52**	**0.29–0.91**	**.02**
Fluent in English (vs not fluent)	**1.71**	**1.07–2.70**	**.03**	1.25	0.72–2.15	.43
More than high school education (ns high school education or less)	1.03	0.65–1.60	.91			
Annual income of $30,000 or more (vs not)	1.67	0.87–3.20	.12			
Employed (vs not)	1.21	0.77–1.90	.41			
Stable housing (vs not)	1.22	0.52–2.80	.65			
3 or more members in household (vs less)	1.32	0.85–2.00	.22			
Comorbidities						
Diabetes	**0.56**	**0.36–0.80**	**.01**	0.73	0.45–1.18	.20
Hypertension	0.56	0.36–0.80	.10			
Hyperlipidemia	0.79	0.51–1.20	.28			
Anxiety	1.36	0.64–2.90	.43			
Depression	0.79	0.47–1.30	.37			
Alcohol intake						
Moderate	1.38	0.70–2.70	.35			
Heavy	1.64	0.97–2.70	.07			

BMI, body mass index.
[a]Bold indicates *P* value < .05.

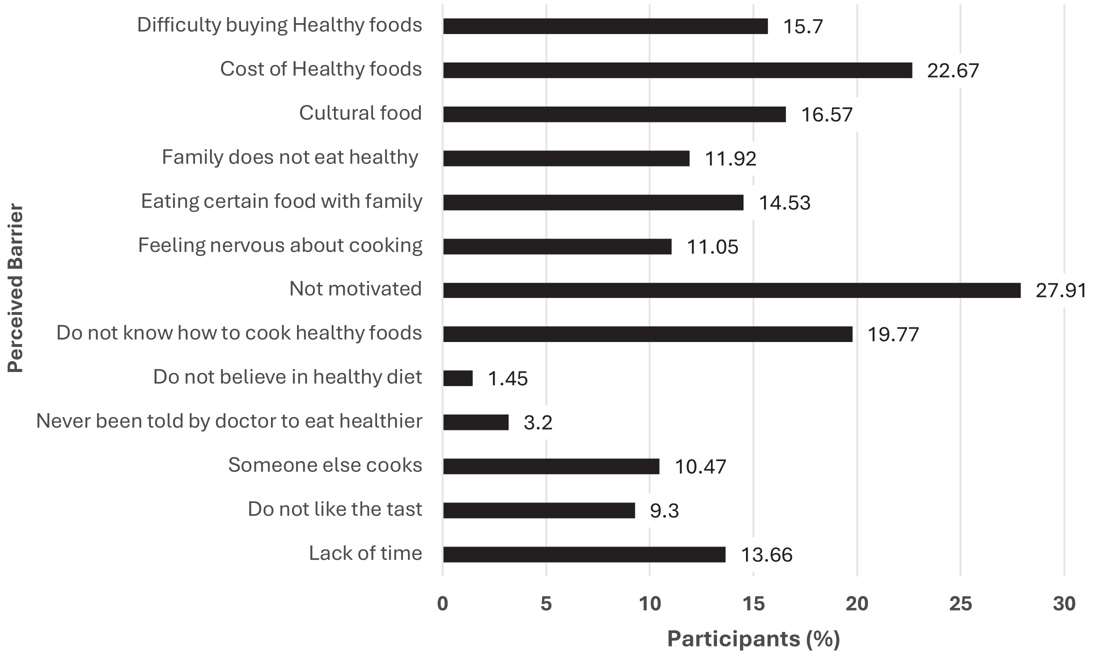

Figure 2. Percentage of participants by the reported perceived barrier to healthy eating.

Table 3. Relationship Between Perceived Barriers and Consumption of Unhealthy Food Categories

	Carbs			Fast food			Fried foods			Sweets			Sugar-sweetened beverages		
Barriers	OR	95% CI	*P* value[a]	OR	95% CI	*P* value	OR	95% CI	*P* value[a]	OR	95% CI	*P* value	OR	95% CI	*P* value[a]
Societal															
Difficulty buying healthy foods	1.48	0.81–2.69	.20	1.34	0.66–2.72	.42	2.22	0.97–5.08	.06	1.87	0.83–4.23	.13	**2.30**	**1.10–4.82**	**.03**
Cost of healthy foods	1.42	0.85–2.38	.18	1.20	0.64–2.27	.57	1.55	0.71–3.42	.27	1.52	0.71–3.23	.28	1.52	0.75–3.06	.25
Community															
Cultural food	**2.97**	**1.55–5.66**	**.001**	1.40	0.70–2.79	.34	**2.44**	**1.09–5.46**	**.03**	1.73	0.77–3.91	.18	**2.13**	**1.01–4.43**	**< .05**
Interpersonal															
Family does not eat healthy	**2.47**	**1.19–5.12**	**.02**	1.77	0.83–3.76	.14	1.75	0.68–4.54	.25	2.29	0.97–5.42	.06	1.80	0.77–4.20	.18
Eating certain food with family	1.55	0.83–2.89	.17	1.94	0.98–3.87	.06	2.05	0.87–4.84	.10	1.16	0.46–2.93	.76	1.37	0.59–3.14	.46
Individual															
Feeling nervous about cooking	1.67	0.82–3.38	.16	1.45	0.65–3.23	.37	**3.00**	**1.23–7.23**	**.02**	1.30	0.47–3.56	.61	**3.37**	**1.53–7.42**	**.003**
Not motivated	1.28	0.80–5.00	.30	**1.79**	**1.01–3.17**	**< .05**	**2.72**	**1.31–5.63**	**.007**	1.46	0.71–3.0	.30	1.40	0.71–2.74	.33
Does not know how to cook healthy	**4.48**	**2.34–8.58**	**< .001**	**2.04**	**1.10–3.80**	**.02**	**2.23**	**1.02–4.85**	**.04**	1.59	0.73–3.47	.24	0.89	0.39–2.01	.78
Do not believe in healthy diet	1.23	0.20–7.48	.82	*b*	*b*	*b*	**6.62**	**1.07–41.16**	**.04**	2.10	0.23–19.34	.51	1.72	0.19–15.76	.48
Never been told by provider to eat healthier	1.45	0.42–5.05	.56	1.71	0.44–6.62	.44	3.79	0.95–15.04	.06	0.83	0.10–6.63	.86	1.54	0.32–7.37	.59
Someone else cooks for me	**3.2**	**1.41–7.23**	**.005**	1.85	0.84–4.07	.13	2.08	0.80–5.44	.14	1.39	0.51–3.83	.52	1.42	0.56–3.64	.46
Do not like the taste	**2.67**	**1.16–6.13**	**.02**	1.28	0.53–3.11	.59	0.97	0.28–3.40	.97	1.21	0.40–3.66	.74	1.30	0.47–3.56	.62
Lack of time	1.54	0.81–2.90	.19	1.65	0.80–3.40	.17	2.23	0.94–5.30	.07	1.26	0.49–3.2	.63	1.00	0.40–2.51	1.00

[a]Bold represents $P < .05$.
[b]All individuals who had fast-food consumption endorsed this barrier.

Discussion

This study evaluated dietary intake and perceived barriers to healthy eating among a diverse and socioeconomically disadvantaged population with SLD. We showed that carbohydrates were the most frequently consumed unhealthy food category; all levels of barriers were associated with higher consumption of unhealthy foods, particularly carbohydrates and fried foods. Those who were older or did any exercise were less likely to have an unhealthy diet. Lack of knowledge and confidence in cooking healthy foods, cultural influence on consumption of certain food groups, and lack of motivation to adhere to recommended lifestyle modifications with diet and exercise were particularly influential in consumption of unhealthy food categories.

This study found that demographic factors did impact diet. Specifically, an increase in age is associated with lower odds of consuming unhealthy foods. Prior studies have highlighted the influence of age on a healthier diet.[26] This may be related to increased knowledge of the role of a healthy diet in managing chronic health conditions that are more prevalent with aging. As noted in prior studies, exercise was also associated with a healthier diet in this study, possibly related to an increase in knowledge or motivation to eat healthier.[19,27,28] Sex and ethnicity also had a trend toward statistical significance ($P = .05$ and $P = .05$, respectively), with female sex and Latino ethnicity having a negative association with unhealthy diet. Studies have shown that women have higher quality diets compared to men.[26,29,30] Discrepancy in diet quality based on sex has been hypothesized to be related to lower knowledge of healthy diets and poorer cooking proficiency.[31] Indeed, in this study, we show that lack of knowledge and confidence in cooking healthy foods was associated with higher consumption of carbohydrates, fast food, and fried foods. With respect to differences across race/ethnicity, it is notable that 82% of the participants were foreign born. As the average American diet is high in processed sugars and saturated fats,[32] foreign born individuals may have healthier diet prior to acculturation to the unhealthy American diet.[33,34]

Barriers at each level of influence resulted in increased rate of unhealthy diet. At the personal level, perceived barriers that yielded multiple categories of unhealthy food consumption included not knowing how to cook healthy foods or feeling nervous or anxious about cooking. These barriers can lead to a decrease in cooking frequency. Studies have found a direct relationship between increased cooking frequency and consumption of healthier food categories, such as vegetables.[35,36] Another perceived personal barrier that influenced consumption of fast food and fried food was lack of motivation to eat healthy. This lack of motivation to eat healthier can be multifactorial: increased temptations and cravings for unhealthy foods[37] or greater reward provided by their taste or satiating effects.[38] One study in an SLD population found that increased motivation to adhere to changes in lifestyle following education was associated with improved SLD outcomes with sustained normalization of liver function tests.[39]

Interpersonal, community, and societal level barriers also influenced consumption of unhealthy food categories. At the interpersonal and community level, family members' dietary habits and the importance of eating cultural foods were in particular associated with higher consumption of carbohydrates, fried foods, and sugar-sweetened beverages. In a qualitative study exploring community partner perspectives on barriers and facilitators to lifestyle modification among Latino and Asian individuals with SLD, misinformation and social influences were cited as barriers, whereas healthy cultural habits and family were identified as facilitators to healthy lifestyle modifications.[40] Considering that most participants in our study were of Latino and Asian ethnic/racial groups, promoting community-based support for healthy cultural dietary habits and family engagement represents an important intervention for reducing unhealthy eating within these communities.[40] At the societal level, difficulty in buying healthy food was associated with increased intake of sugary drinks. This potentially reflects ease of access to sugar-sweetened beverages in this country.[41] In a recent study in California, a societal intervention imposing a tax on sugar-sweetened beverages resulted in a significant reduction in the purchasing of these drinks.[42] In our study, nearly 24.1% of participants with difficulty buying healthy food also cited cost of food as a barrier. Although the cost of healthy foods had a positive relationship with higher consumption of all unhealthy food categories, this was not statistically significant. On the other hand, difficulty buying healthy food, reflecting food insecurity, was associated with nearly 2.5 times higher odds of sugar-sweetened beverage intake. This suggests that aside from raising cost of sugar-sweetened beverages, improving access to healthy foods may play a greater role in reducing sugar-sweetened beverage consumption among socioeconomically disadvantaged populations.

This study has a few limitations. First, participants were recruited in a single-center safety-net health-care system, and the results may not be generalizable to other health-care settings. Second, the relationship between unhealthy diet consumption and the clinical outcomes of SLD was not assessed. However, a recent study evaluating the clinical outcomes following SLD education in this cohort found that posteducation motivation for lifestyle change was the most significant driver of the desired clinical outcome of sustained alanine aminotransferase normalization.[39] Additionally, dietary surveys can be subject to recall bias. Lack of motivation to eat healthy and exercise was the most commonly reported barrier to lifestyle change in our study and was associated with certain unhealthy food categories suggesting an indirect link between unhealthy eating and clinical outcomes. Lastly, there may be unmeasured factors that influence consumption of unhealthy diet. Nevertheless, major strengths of this study include the understudied racially/ethnically diverse and socioeconomically disadvantaged SLD safety-net population, with comprehensive assessment of perceived barriers at different levels of influence and factors that impact unhealthy diet frequency.

Conclusion

In summary, we identified perceived barriers to a healthy diet in SLD across personal, interpersonal, community, and societal levels. This suggests that interventions directed at addressing any or multiple levels of influence is likely to promote healthy eating, especially in younger age groups and those with limited physical activity. Our findings also highlight that providing culturally tailored education on recommended healthy foods and cooking practices is critical for enhancing confidence and motivation for dietary change. Ultimately, incorporating this approach may increase adherence to dietary recommendations in SLD.

Supplementary Materials

Material associated with this article can be found, in the online version, at https://doi.org/10.1016/j.gastha.2026.101024.

References

1. Israelsen M, Francque S, Tsochatzis EA, et al. Steatotic liver disease. Lancet 2024;404:1761–1778.
2. Ho GJK, Tan FXN, Sasikumar NA, et al. High global prevalence of steatotic liver disease and associated subtypes: a meta-analysis. Clin Gastroenterol Hepatol 2025;23:2423–2432.e1.
3. Ciardullo S, Carbone M, Invernizzi P, et al. Exploring the landscape of steatotic liver disease in the general US population. Liver Int 2023;43:2425–2433.
4. Lee BP, Dodge JL, Terrault NA. National prevalence estimates for steatotic liver disease and subclassifications using consensus nomenclature. Hepatology 2024;79:666–673.
5. Kardashian A, Serper M, Terrault N, et al. Health disparities in chronic liver disease. Hepatology 2023;77:1382–1403.
6. Xiao Y, Zhang X, Yi D, et al. Mediterranean diet affects the metabolic outcome of metabolic dysfunction-associated fatty liver disease. Front Nutr 2023;10:1225946.
7. Tapper EB, Mehta M, Leung CW. Food insecurity is associated with chronic liver disease among US adults. J Clin Gastroenterol 2023;57:737–742.
8. Lee JY, Kim S, Lee Y, et al. Higher adherence to the mediterranean diet is associated with a lower risk of steatotic, alcohol-related, and metabolic dysfunction-associated steatotic liver disease: a retrospective analysis. Nutrients 2024;16:3551.
9. Rinaldi L, Pafundi PC, Galiero R, et al. Mechanisms of non-alcoholic fatty liver disease in the metabolic syndrome. A narrative review. Antioxidants (Basel) 2021;10:270.
10. Berna G, Romero-Gomez M. The role of nutrition in non-alcoholic fatty liver disease: pathophysiology and management. Liver Int 2020;40(suppl 1):102–108.
11. Beck AL, Iturralde E, Haya-Fisher J, et al. Barriers and facilitators to healthy eating among low-income Latino adolescents. Appetite 2019;138:215–222.
12. Lima JPM, Costa SA, Brandao TRS, et al. Food consumption determinants and barriers for healthy eating at the Workplace-A university setting. Foods 2021;10:695.
13. Miller KC, Geyer B, Alexopoulos AS, et al. Disparities in metabolic dysfunction-associated steatotic liver disease prevalence, diagnosis, treatment, and outcomes: a narrative review. Dig Dis Sci 2025;70:154–167.
14. Chuang E, Yu S, Georgia A, et al. A decade of studying drivers of disparities in end-of-life care for black Americans: using the NIMHD framework for health disparities research to map the path ahead. J Pain Symptom Manage 2022;64:e43–e52.
15. Alvidrez J, Castille D, Laude-Sharp M, et al. The National Institute on Minority Health and Health Disparities Research Framework. Am J Public Health 2019;109:S16–S20.
16. Patel S, Partida D, Magee C, et al. Steatotic liver disease education enhances knowledge and confidence to adhere to provider recommendations in diverse and vulnerable populations. Gastro Hep Adv 2025;4:100589.
17. Mukhtar NA, Evon DM, Yim C, et al. Patient knowledge, beliefs and barriers to hepatitis B care: results of a multicenter, multiethnic patient survey. Dig Dis Sci 2021;66:434–441.
18. Bastani R, Glenn BA, Taylor VM, et al. Integrating theory into community interventions to reduce liver cancer disparities: the health behavior framework. Prev Med 2010;50:63–67.
19. Patel S, Kim RG, Shui AM, et al. Fatty liver education promotes physical activity in vulnerable groups, including those with unhealthy alcohol use. Gastro Hep Adv 2024;3:84–94.
20. Pinho MGM, Mackenbach JD, Charreire H, et al. Exploring the relationship between perceived barriers to healthy eating and dietary behaviours in European adults. Eur J Nutr 2018;57:1761–1770.
21. Herren OM, Gillman AS, Marshall VJ, et al. Understanding the changing landscape of health disparities in chronic liver diseases and liver cancer. Gastro Hep Adv 2023;2:505–520.
22. Consultation WHOE. Appropriate body-mass index for Asian populations and its implications for policy and intervention strategies. Lancet 2004;363:157–163.
23. Alberti KG, Eckel RH, Grundy SM, et al. Harmonizing the Metabolic Syndrome: a Joint Interim Statement of the International Diabetes Federation task force on epidemiology and prevention; National Heart, Lung, and Blood Institute; American Heart Association; World Heart Federation; International Atherosclerosis Society; and International Association for the Study of Obesity. Circulation 2009;120:1640–1645.
24. Vittinghoff E, McCulloch CE. Relaxing the rule of ten events per variable in logistic and cox regression. Am J Epidemiol 2007;165:710–718.
25. Scrucca L, Fop M, Murphy TB, et al. Mclust 5: clustering, classification and density estimation using gaussian finite mixture models. R J 2016;8:289–317.
26. Imamura F, Micha R, Khatibzadeh S, et al. Dietary quality among men and women in 187 countries in 1990 and 2010: a systematic assessment. Lancet Glob Health 2015;3:e132–e142.

27. Joo J, Williamson SA, Vazquez AI, et al. The influence of 15-week exercise training on dietary patterns among young adults. Int J Obes (Lond) 2019;43:1681–1690.
28. Tucker M, Reicks M. Exercise as a gateway behavior for healthful eating among older adults: an exploratory study. J Nutr Educ Behav 2002;34(suppl 1):S14–S19.
29. Overcash F, Reicks M. Diet quality and eating practices among Hispanic/Latino men and women: NHANES 2011-2016. Int J Environ Res Public Health 2021; 18:1302.
30. Maskarinec G, Namatame LA, Kang M, et al. Differences in the association of diet quality with body fat distribution between men and women. Eur J Clin Nutr 2020; 74:1434–1441.
31. Zhao H, Andreyeva T. Diet quality and health in older Americans. Nutrients 2022;14:1198.
32. Grant A, Dash C, Adams-Campbell LL. The association between diet quality and metabolic syndrome among older African American women. Nutrients 2024;16:3040.
33. Arandia G, Sotres-Alvarez D, Siega-Riz AM, et al. Associations between acculturation, ethnic identity, and diet quality among U.S. Hispanic/Latino Youth: findings from the HCHS/SOL Youth study. Appetite 2018; 129:25–36.
34. Vo V, Lopez G, Malay S, et al. Cardiovascular risk factors among Asian Americans: perspectives on the role of acculturation in cardiovascular diseases health disparities. J Immigr Minor Health 2024;26:409–420.
35. Farmer N, Lee LJ, Powell-Wiley TM, et al. Cooking frequency and perception of diet among US adults are associated with US healthy and healthy mediterranean-style dietary related classes: a latent class profile analysis. Nutrients 2020;12:3268.
36. Mills S, White M, Brown H, et al. Health and social determinants and outcomes of home cooking: a systematic review of observational studies. Appetite 2017; 111:116–134.
37. Werthmann J, Tuschen-Caffier B, Strobele L, et al. Healthy cravings? Impact of imagined healthy food consumption on craving for healthy foods and motivation to eat healthily - results of an initial experimental study. Appetite 2023;183:106458.
38. Leng G, Adan RAH, Belot M, et al. The determinants of food choice. Proc Nutr Soc 2017;76:316–327.
39. Rebecca G, Kim SP, Shui AM, et al. Clinical outcomes following a patient-centered educational intervention on steatotic liver disease: a comparison of vulnerable patients with and without diabetes. Gastro Hep Adv 2025;4:100710.
40. Tsuchiyose E, Salimi A, Magee C, et al. Culturally tailored steatotic liver disease management: Latino and Asian community partner perspectives and recommendations. Hepatol Commun 2025;9:e0749.
41. Costa D, Warkentin S, Oliveira A. Sugar-sweetened beverages, effects on appetite and public health strategies to reduce the consumption among children: a review. Porto Biomed J 2022;7:e172.
42. White JS, Basu S, Kaplan S, et al. Evaluation of the sugar-sweetened beverage tax in Oakland, United States, 2015-2019: a quasi-experimental and cost-effectiveness study. PLoS Med 2023;20:e1004212.

Received September 27, 2025. Accepted May 20, 2026.

Correspondence:
Address correspondence to: Mandana Khalili, MD, University of California San Francisco, San Francisco General Hospital, 1001 Potrero Ave, Building 5, Suite 3D, San Francisco, California 94110. e-mail: Mandana.Khalili@ucsf.edu.

Authors' Contributions:
Serena N. Gilmore: Designed the study, participated in statistical analysis and interpretation, wrote the manuscript, and approved final submission. Catherine Magee: Performed the study, collected data, and reviewed and approved the final manuscript. Mandana Khalili: Designed the study, provided material support, performed statistical analyses, edited the manuscript, and approved final submission.

Conflicts of Interest:
The author discloses the following: Mandana Khalili is a recipient of research grants (to her institution) from Gilead Sciences and Intercept Pharmaceuticals, and she has served as a consultant for Gilead Sciences Inc, GlaxoSmithKline Pharmaceuticals, and Resolution Therapeutics. The remaining authors disclose no conflicts.

Funding:
This work was in part supported by National Institutes of Health, K24AA022523 (Mandana Khalili) and in part by the San Francisco Cancer Initiative (SF CAN), a collaborative community effort initiated by the UCSF Helen Diller Family Comprehensive Cancer Center to reduce the cancer burden across San Francisco and beyond (https://www.sfcancer.org).

Ethical Statement:
This study was approved by the Institutional Review Board of the University of California, San Francisco (IRB #19–27625) and Zuckerberg San Francisco General Hospital.

Data Transparency Statement:
Data, analytic methods, and study materials can be made available to other researchers in this manuscript upon reasonable request.

Reporting Guidelines:
Reporting Guidelines were not applicable for this article type.

Gastro Hep Advances, Volume *5*

Supplemental information

Perceived Barriers to a Healthy Diet and Factors Associated With Unhealthy Food Consumption in Steatotic Liver Disease

Serena N. Gilmore, Catherine Magee, and Mandana Khalili

Perceived Barriers to a Healthy Diet and Factors Associated with Unhealthy Food Consumption in Steatotic Liver Disease

Serena N Gilmore, Catherine Magee, and Mandana Khalili

Table of contents

Table of Contents

SUPPLEMENTAL TABLE 1. 2

SUPPLEMENT TABLE 2.......... 4

Table S1. Frequency of consumption of unhealthy food categories survey questionnaire

Question	Response choices
**How often do you eat rice/bread/tortillas?**	None<br>1 time per week<br>2-3 times per week<br>4-5 times per week<br>6 or more times per week
**On average, how often do you eat fast food?**	Not at all<br>One time per month<br>2-3 times per month<br>4-5 times per month<br>6 or more times per month
**How often do you eat fried food? (chow mein, fried chicken, chips, french fries, chicharrones, tostadas, refried beans)**	None<br>1 time per week<br>2-3 times per week<br>4-5 times per week<br>6 or more times per week
**How often do you have sweets such as baked goods, candy, and ice cream?**	None<br>1 time per week<br>2-3 times per week<br>4-5 times per week<br>6 or more times per week
**How often do you drink sugar-sweetened beverages per week? (ie: soda, boba, agua fresca,<br>juice, sports drink, etc)**	None<br>1 time per week<br>2-3 times per week<br>4-5 times per week<br>6 or more times per week
**How many cups of tea (black or green) did you typically drink in the past year? (one cup oftea equals 8 ounces, hot or cold)**	None<br>Occasionally (less than 1 per day)<br>1 per day<br>2 per day<br>3 or 4 per day<br>More than 4 per day
**How many cups of coffee did you typically drink in the past year? (one cup of coffee equals 8 ounces. Count one espresso as one cup regardless of size)**	None<br>Occasionally (less than 1 per day)<br>1 per day<br>2 per day<br>3 or 4 per day<br>More than 4 per day

**Do you drink your coffee or tea with sugar?**	Yes<br>Type of sweetener:<br>Sugar (raw/brown/refined etc.)<br>Artificial sweetener (Splenda, Sweet'N Low, Equal, Stevia etc.)<br>Sweetened Creamer (Coffee Mate, Delight, etc)<br>Honey/agave<br>Number of packets or spoons: _____________<br>No

Table S2. Survey question regarding barriers to eating healthy foods

**Level of Influence**	**What barriers (if any) do you experience in following your provider's recommendations for healthy eating?**
Societal	-Difficulty buying healthy foods<br>-Cost of healthy foods<br>-Difficulty buying healthy foods
Community	-In my culture we eat certain foods, and it is important for me to eat this way with other members of my community
Interpersonal	-Family members do not eat healthy, so difficult for me to change my diet<br>-In my family, we eat certain foods, and it is important for me to eat this way with my family
Individual	-Feeling nervous or anxious about cooking<br>-Not motivated to eat healthy foods/exercise<br>-Do not know how to cook healthy foods<br>-Do not believe healthy diet/exercise are important<br>-Never been told by doctor to eat healthier/exercise more<br>-Some else cooks for me<br>-Do not like the taste of healthy foods<br>-Lack of time to eat healthy foods/exercise
